# Melatonin induces drought stress tolerance by regulating the physiological mechanisms, antioxidant enzymes, and leaf structural modifications in *Rosa centifolia* L

**DOI:** 10.1016/j.heliyon.2024.e41236

**Published:** 2024-12-17

**Authors:** Muhammad Ahsan, Adnan Younis, Aftab Jamal, Mohammed O. Alshaharni, Uthman Balgith Algopishi, Abeer Al-Andal, Mateen Sajid, Muhammad Naeem, Jawad Ahmad Khan, Emanuele Radicetti, Mohammad Valipour, Gulzar Akhtar

**Affiliations:** a Department of Horticultural Sciences, The Islamia University of Bahawalpur, Bahawalpur, 63100, Pakistan; b Institute of Horticultural Sciences, University of Agriculture, Faisalabad, 38040, Pakistan; c Department of Soil and Environmental Sciences, Faculty of Crop Production Sciences, The University of Agriculture, Peshawar, 25130, Pakistan; d Biology Department, College of Science, King Khalid University, Abha, 61321, Saudi Arabia; e Department of Biology, College of Science, King Khalid University, Abha, 61413, Saudi Arabia; f Department of Horticulture, Ghazi University, Dera Ghazi Khan, 32200, Pakistan; g Department of Pharmacy, Shah Abdul Latif University Khairpur, 66111, Pakistan; h Department of Chemical, Pharmaceutical and Agricultural Sciences (DOCPAS), University of Ferrara, 44121, Ferrara, Italy; i Department of Engineering and Engineering Technology, Metropolitan State University of Denver, Denver, CO, 80217, USA; j Department of Horticulture, Muhammad Nawaz Shareef University of Agriculture, Multan, 66000, Pakistan

**Keywords:** Enzyme activity, Photosynthesis, Proline, Sustainable floriculture, Water deficiency

## Abstract

Melatonin is considered an effective bio-stimulant that is crucial in managing several abiotic stresses including drought. However, its potential mechanisms against drought stress in fragrant roses are not well understood. Here, we aim to investigate the role of melatonin on *Rosa centifolia* plants cultivated under drought stress (40 % field capacity) and normal irrigation (80 % field capacity). Plant growth traits, gaseous exchange, antioxidants, osmolytes, oxidative stress, and leaf anatomical attributes were measured. All pots were arranged with a completely randomized design with two-factor factorial setup. Foliar application of melatonin was carried out on the next day of drought treatment and was repeated weekly, while normal watering was regarded as control. Drought stress significantly enhanced oxidative stress markers and reduced growth parameters in water-deficit rose plants. However, melatonin spray (100 μM) produced increased plant height (16 %), flower yield (16 %), petal fresh and dry biomass (7 % and 38 %), total chlorophyll (48 %), contents of carotenoid (54 %), and gaseous exchange traits such as stomatal conductance (25 %), photosynthetic rate (91 %), and transpiration rate (3 %), in water-deficient plants. Likewise, the accretion of catalase, superoxide dismutase, soluble protein, proline, and glycine betaine contents was recorded by 22 %, 45 %, 58 %, 7 %, and 6 %, respectively, in drought-stressed plants, due to melatonin treatment. Increment of oxidative stress indicators i.e. malondialdehyde (−37 %) and hydrogen peroxide (−27 %) was diminished by melatonin triggered by drought stress. Furthermore, leaf cortex (51 %), vascular bundle area (76 %), palisade cell area (59 %), and lamina thickness (42 %) were remarkably increased with melatonin foliar sprays in water-deficit plants. The results of this study recommend that melatonin is a protective agent against drought stress and has potential application prospects in the rose-producing regions suffering from water deficiency. Future studies should focus on molecular responses of *R. centifolia* to drought stress to further develop stress alleviation strategies in floricultural crops.

## Graphical abstract

**Figure:**
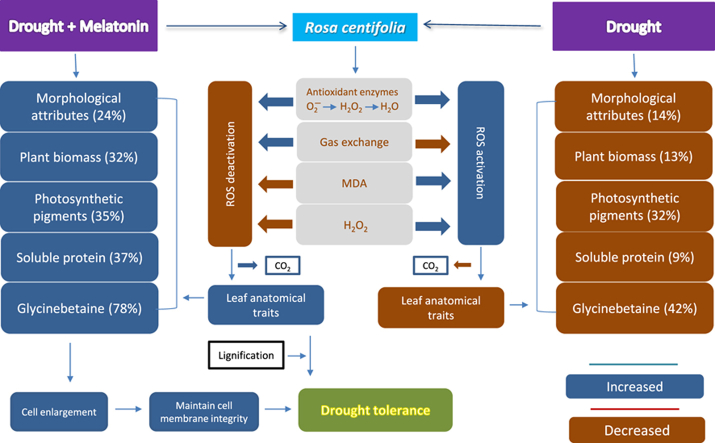

## 1 Introduction

Water is the foundation of life on our planet, vital for growth and the existence of all living organisms, including plants [1]. Water shortage in the rooting medium, designated as drought stress, typically results in different plant metabolic and physiological disorders [2]. Drought is one of the most destructive environmental stresses and will lessen plant yield by 31 % by 2028, then presently obtained crop yield [3]. According to Toscano and Romano [4], southern Europe, southern Asia, and southern and northern Africa are expected to be mostly interrupted by 30 % more drylands, drier summers, and less rainfall. Water deficiency can reduce plant growth by disturbing photosynthetic pigments and plant biomass, which leads to a decrease in carbon assimilation and plant yield [5]. Drought-induced lipid peroxidation in plant tissues leads to the production of reactive oxygen species (ROS), which are responsible for damage to DNA and protein and disturb the structure and functions of plant cell membranes [6]. Higher ROS production is involved in the initiation of stress-induced biochemical, molecular, and physiological responses [7]. Abscisic acid, a crucial stress signaling component, is produced under drought stress, and it can regulate gene expression for biochemical responses by producing antioxidant enzymes [8]. Severe water stress can lead to a cascade of oxidative injury that ultimately results in partial or complete senescence of plants [9]. Generally, plant hormones normalize the growth of plants and increase resistance, hence, recognizing the possible growth promotors and their mechanism is critical for the consolidation of agricultural drought tolerance [10].

Melatonin (N-acetyl-5-methoxytryptamine) is well-thought-out a unique plant growth stimulant. It is an excellent antioxidant molecule with the substantial potential to scavenge reactive oxygen species and improve abiotic stress tolerance [11,12]. Melatonin has been concerned with numerous physiological functions encompassing regulation of growth and development of plants, rooting, osmo-protectants, photosynthesis, delay in leaf and flower senescence as well as controlling physiological mechanisms by encouraging differential gene expression [13]. It can boost secondary metabolites synthesis by its interaction with reactive oxygen species and reactive nitrogen species, generating a chain reaction that defuses several toxic oxygen derivatives, thus melatonin can be regarded as a free radical scavenger [14]. By acting as an antioxidant for nitrogen species and enabling oxygen to be metabolically tolerated, this melatonin helps to reduce damage caused by drought stress [15]. Furthermore, melatonin protects the macromolecules present in the cells from damage by cell enlargement and rhizogenesis at the tissue level which leads to improved physicochemical processes in the plants [10]. It was observed that melatonin application increases photosynthetic rate, enhances stomatal conductance, and maintains the stability of the membrane due to higher antioxidant activity [16,17]. Similarly, melatonin enhanced drought stress tolerance in *Solanum lycopersicum*, *Cumumis sativus*, and *Nicotiana benthamine* plants [10,18,19]. So, the positive role of melatonin under water-deficit conditions has been reported mainly in herbaceous plant species. Though, there are some studies showing the impact of melatonin on drought tolerance at biochemical and physiological levels, no work on anatomical alteration in woody plants is reported, and since their action depends on the mode of supplementation and the plant species.

Rose is considered as the queen of flowers which represents the foremost ornamental plant in the floricultural industry worldwide. It is a perennial flowering woody plant that belongs to the Rosaceae family. Genus *“Rosa”* comprises approximately 20,000 cultivars and more than 200 species across the world [20]. Various *Rosa* species are cultivated for numerous purposes such as indoor and garden plantation; cut flower business and occasionally food. *Rosa centifolia* is one of the four species that are used for essential oil extraction in Pakistan [21]. However, roses are highly vulnerable to different abiotic stresses during the active production stage, among which water shortage is a vital stress factor that disturbs the yield and quality of flowers [22]. Previous investigations have shown the role of different bio-stimulants on water-deficiency tolerance in some plant species. However, to the best of our knowledge, no work is available regarding the role of melatonin in floral characteristics, anatomical attributes, and oxidative stress-linked parameters in fragrant roses especially *R. centifolia* under water-stressed conditions.

To reduce this research gap, we hypothesize that exogenous melatonin treatment positively improves the morpho-physiological, biochemical, and leaf anatomical attributes of *R. centifolia* under water deficit conditions. The specific objectives of this study are: (i) to investigate the impacts of melatonin in boosting drought stress resistance and to study the causal mechanism through which melatonin enhances drought stress tolerance; (ii) to study the role of melatonin in on the growth and performance of reactive oxygen species scavenging photosynthetic machinery, activities of antioxidant, and the potential variations in leaf architecture in *R. centifolia* to reduce oxidative damage under drought-stressed conditions. These results provide some insights for ameliorating the drought impacts on the growth performance and quality of elegant *R. centifolia* and may offer a strategy for encouraging rose cultivation in water deficit regions.

## 2 Materials and methods

### 2.1 Experimental site and planting materials

An outdoor pot experiment was carried out from mid-September 2021 to late April 2022 at the Horticultural Research Area (31° 25′ 42″ N, 73° 09′ 34″ E, 300 m above sea level) at the University of Agriculture, Faisalabad, Pakistan. The climate of the research area is semi-arid with scarce annual rainfall (Table 1). Two-year-old vigorous and healthy cuttings of *R. centifolia* were collected from the Rose Project of the university. These cuttings (20 cm long) were planted in terracotta pots having a depth of 35 cm and 24 cm diameter containing sandy clay loam soil (single plant in each pot). The soil's pH was 7.54 and its electrical conductivity was 1.19 dS m^−1^. Soil nutrient status were: nitrogen 154.4 mg kg^−1^; phosphorus 11.18 mg kg^−1^ and potassium 103.02 mg kg^−1^. The pots in the experiment were arranged according to a completely randomized design with six replications, based on a two-factor factorial setup. Basal doses of nitrogen, phosphorus, and potassium (10 g pot^−1^) were manually applied using 46 % urea, 18 % single super phosphate, and 50 % muriate of potash (Fauji Fertilizer Company Limited, Pakistan). The second and third doses of these fertilizers were applied at 20 d and 40 d after sprouting of the cuttings. The other management practices like weeding and pesticide application were the same for all treatments.

**Table 1:** The weather conditions of experimental site during the study period.

Months	Mean Temp. (°C)		Relative Humidity (%)	Light period (h)	Dark period (h)
	Max.	Min.			
September	29.3	14.2	65.6	11.5	12.5
October	26.4	12.6	62.1	11.1	12.9
November	21.1	9.7	58.2	10.5	13.5
December	16.1	8. 0	59.1	10.1	13.9
January	15.2	7.3	51.3	10.4	13.6
February	16.7	10.5	53.7	11.2	12.8
March	24.6	13.0	59.2	12.6	11.4
April	27.2	15.4	62.0	13.2	10.8

### 2.2 Experimental treatments

The study included four treatments i.e., N for normal conditions with 80 % field capacity; N + MT for foliar supplementation of melatonin with 80 % field capacity; D for drought stress (40 % field capacity); and D + MT for drought stress (40 % field capacity) and melatonin. Each treatment possessed ten pots; there were 3.8 kg of soil in each pot. The melatonin (Sigma-Aldrich Chemie, Steinheim, Germany) at 100 μM was prepared by dissolving melatonin in ethanol and used for foliar spray [10]. The screening of rose species, concentration of melatonin, and selection of field capacities was done on a fresh weight basis. It was recorded that when different rose species were treated with varying contents of melatonin (1–200 μM), the 100 μM of melatonin maximally improved the growth of the *R. centifolia* in terms of fresh weight. Similarly, significant differences in fresh weight were recorded at 40 % field capacity and 80 % field capacity. The first foliar spray of melatonin was done on next day after the application of drought-stress treatment at 5 leaf stage of the plants. A total of five sprays were applied in five weeks. The foliar spray of melatonin was carried out in the early morning (06:00 to 7:00 a.m.) with the help of a compression layer with a capacity of 1 L. To avoid contamination, a polyethylene sheet was used to cover the top of each pot before the application of each foliar spray. Additionally, to avoid the loss of moisture through pot's base hole, all the pots, except control plants, were placed inside white polythene bags. The estimation of water application in soil was done by a soil humidity meter (ML3 ThetaProbe, Delta-T Devices Co., Burwell, UK). Throughout the study period (around six months), the pots received daily irrigations in a specified field capacity and weighed on a digital portable balance to maintain the required field capacity levels. Every irrigation for normal-watered plants received 60 ± 2 mm water, whereas drought-stressed plants consisted of 30 ± 2 mm water for each irrigation.

### 2.3 Quantification of morphological attributes

Plant physical attributes, like the number of leaves branch^−1^, flower numbers, and number of petals flower^−1^ were noted after flowers were fully opened. The height of the plant (cm) and diameter of the flowers (mm) were estimated by measuring tape and digital caliper, respectively. There were 12 replicates for each measurement of the morphological traits. The leaf area (mm^2^) was measured with a leaf area meter (Delta-T, Ltd., Cambridge, UK). Fresh and dry weights of flower petals (g) were measured according to Celikel et al. [23] by using all petals of entire replicates from a single flower at individual tests.

### 2.4 Estimation of photosynthetic parameters

To estimate the photosynthetic characteristics i.e., stomatal conductance (*gs*), net photosynthetic rate (*A*), and transpiration rate (*E*), an infrared gas analyzer (LCA-4, Analytical Development Co. Hoddesdon, UK) was employed. The first reading (total of 8 readings) of these parameters was collected three weeks after the application of the last foliar melatonin spray between 8:00 a.m. to 10:00 a.m. in sunlight assuming the plants were fully functional at that time [24]. The data for stomatal conductance were recorded in mmol m m^−2^ S^−1^, whereas, net photosynthetic rate was measured in μmol m^−2^ S^−1^.

### 2.5 Detection of pigments

After three weeks of the final melatonin foliar spray, fresh leaf tissues (1.0 g) were taken from each treatment and cut into pieces of 0.5 cm. For the assessment of total chlorophyll (Chl_t_) and carotenoid (CAR), the extraction was done overnight at 4 °C in 80 % acetone (10 mL) by following the protocols suggested by Arnon [25], and Davies [26], respectively. The data was collected from mean of 8 readings of each treatment. Total chlorophyll contents were recorded in mg g^−1^, while carotenoid contents were recorded in μg g^−1^ on a fresh weight basis.

### 2.6 Determination of antioxidants enzymatic activities

One month after the application of the last melatonin spray, the first reading of fresh leaf tissues (total of 8 readings) weighing approximately 1.0 g was normalized in 50 mM phosphate buffer and dithiothreitol (DTT) and centrifuged at 4 °C for 20 min at 12000×*g* according to the methodology proposed by Dixit et al. [27]. The supernatant was detached, and the readings for activities of antioxidant enzymes at different wavelengths were noted by using a spectrophotometer (Jenway, Staffordshire, UK). Zhang et al. [28] technique was followed to determine the catalase and peroxidase activities, and absorbance was noted at 240 nm and 470 nm, respectively. The methods described by Cakmak [29] and Giannopolitis and Ries [30] were used to measure the ascorbate peroxidase and superoxide dismutase, respectively. The activity of ascorbate peroxidase was noted at 290 nm while superoxide dismutase activity was observed at 560 nm. All data catalase, peroxidase, and superoxide dismutase were observed in min^−1^ g^−1^ fresh weight basis, whereas, ascorbate peroxidase activity was measured in ABA digested g^−1^ of fresh weight h^−1^.

### 2.7 Estimation of glycinebetaine, total soluble protein, and free proline

Glycinebetaine contents were calculated by taking 0.5 g of dry leaf tissue in 10 mL of toluene (0.5 %) and letting it sit at 4 °C for an entire night. The filtrate was combined with H_2_SO_4_ in an amount of 1.0 mL. This extract (0.5 mL) was put into a test tube with 200 μL of a KI_3_ solution, and everything was then chilled in a chiller. Then, 1–2 di-chloroethane (5.0 mL) and ice-cooled deionized water (2.8 mL each) were added. Using a spectrophotometer, the organic layer's absorbance at *λ* 365 nm was measured. By using a curve and following the procedure of Grieve and Grattan [31], the glycinebetaine contents were recorded. The mean values of glycinebetaine were recorded from 8 readings in terms of μmol g^−1^ on a fresh weight basis. Furthermore, about 0.5 g of fresh leaf tissues were taken and extracted with 10 mL of 50 mM potassium phosphate to buffer in an ice bath to measure the total soluble protein content. For 15 min at 4 °C, the aliquot was centrifuged at 10,000×*g*. The mean soluble protein levels in the extract were calculated by following Bradford [32] from 8 readings and expressed in mg mL^−1^ fresh weight. Additionally, a mixture of fresh leaf tissues (1.0 g) was taken in 3 % aqueous sulfosalicylic acid (5 mL) to measure the free proline levels using a spectrophotometer (Jenway, Staffordshire, UK), as suggested by Ahmad et al. [33] and expressed in μmol g^−1^ on a fresh weight basis.

### 2.8 Malondialdehyde and hydrogen peroxide

Malondialdehyde was measured by extracting fresh leaf tissues (0.5 g) in 5 mL of 1 % trichloroacetic acid (*w/v*), as explained by Li et al. [34], and mean values were noted from 8 readings and expressed in nmol g^−1^ fresh weight. For the estimation of hydrogen peroxide, 0.5 g of fresh leaf tissue was crushed with 5 mL of 0.1 % (*w/v*) trichloroacetic acid (TCA) in a cooled mortar. We centrifuged the mixture at 12,000×*g* for 15 min. Following the blend's vortexing, the optical density was measured at 390 nm, and hydrogen peroxide was calculated using the methodology of Velikova et al. [35] for 8 times and mean values were expressed in terms of μmol hydrogen peroxide scavenged g^−1^ fresh weight.

### 2.9 Measurement of leaf anatomical features

The anatomical study of the leaves of *R. centifolia* was conducted by free-hand sectioning. The one cm piece was cut from the leaf center along with the midrib. For a few days, a solution of formalin-acetic acid was utilized for preservation [36]. A solution of acetic acid (one-part acetic acid and three-part ethyl alcohol) was utilized for long-term preservation. All samples were prepared and fixed in formalin acetic acid solution, which was composed of distilled water (10 %), acetic acid (5 %), formalin (10 %), and ethyl alcohol (50 %). Following Sass [37], the sectioning, staining, and mounting procedures were carried out. A light microscope (Nikon 104, Japan) was used to examine 4 slides of each treatment for every parameter.

### 2.10 Statistical analysis

The STATISTIX (version 8.1) program was used to statistically analyze the collected data and carry out an analysis of variance methodology. The least significant difference test at the 5 % level of probability was used to compare treatment means. Principal component analysis and a correlation matrix of various morpho-physiological, biochemical, and leaf anatomical traits of *R. centifolia* under normal watered and drought stress conditions were estimated using OriginPro Software, (Origin Lab Corporation, Northampton, MA, USA) version 2024.

## 3 Results

### 3.1 Plant morphological characteristics

**Fig. 1:**
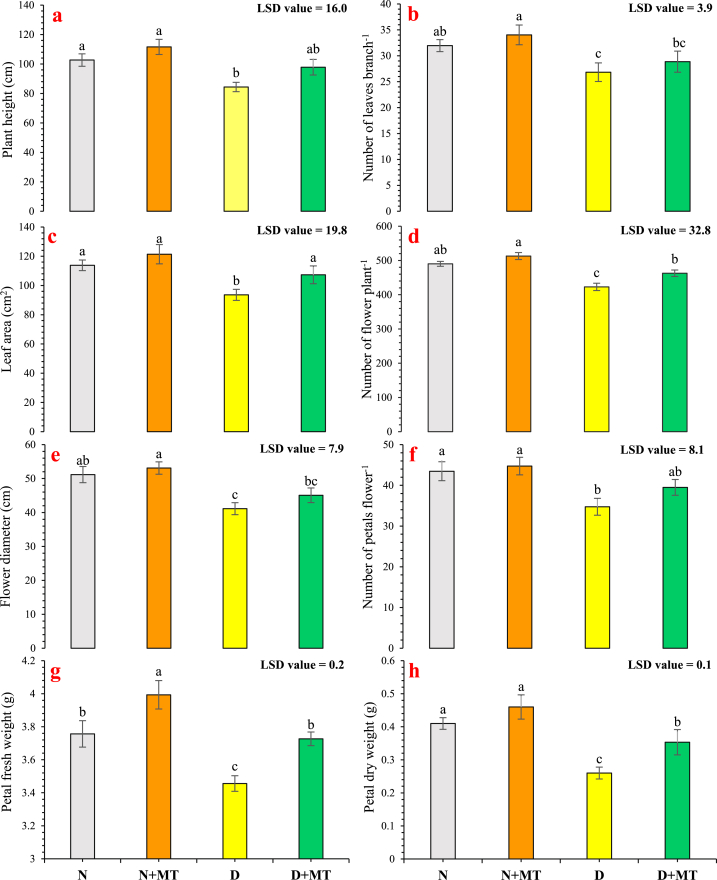
Impact of melatonin on the plant length (**a**), the leaves number branch^−1^ (**b**), the area of leaf (**c**), the number of flower plant^−1^ (**d**), the flower diameter (**e**), the number of petals flower^−1^ (**f**), petal fresh weight (**g**), petal dry weight (**h**) of *Rosa centifolia* under drought-stressed conditions. A mean of six replicates standard error are displayed in each bar. After using the least significant difference (LSD) test, alphabetical lettering that is different from one another shows statistically significant variations at *p* < 0.05. N = normal water; MT = melatonin; D = drought stress.

Statistically, the supplementation of melatonin significantly (*p* < 0.05) enhanced the plant height of *R. centifolia* by 16 % under drought-stressed conditions and by 8.7 % with normal conditions (control). Drought stress (40 % field capacity) markedly reduced plant height (−17.8 %) to normal watered plants (Fig. 1a). Similarly, leaf numbers and their area of *R. centifolia* leaves were significantly (*p* < 0.05) increased by 7.6 % and 14.7 %, respectively, as melatonin application enhanced both leaf attributes under drought-stressed conditions (Fig. 1b and c). Water-deficit conditions remarkably reduced flower numbers, diameter, and the number of petals flower^−1^ of the roses by 8.6 %, 19.6 %, and 20.1 %, respectively, as compared to the control treatment. The melatonin foliar spray increased these floral attributes by 7.3 %, 3.8 %, and 2.9 % under normal-water conditions and by 15.9 %, 9.6 %, and 13.7 % under water deficit conditions, respectively (Fig. 1d–f). Drought stress reduced significantly (*p* < 0.05) petal fresh weight (8 %) and dry weight (36.6 %) in this study. The melatonin applied by foliar means increased fresh and dry petal biomass by 6.3 % and 12.2 %, in controlled conditions and by 7.8 % and 35.9 %, respectively, in water-stress conditions (Fig. 1g and h).

### 3.2 Photosynthetic characteristics, leaf total chlorophyll, and carotenoid

**Fig. 2:**
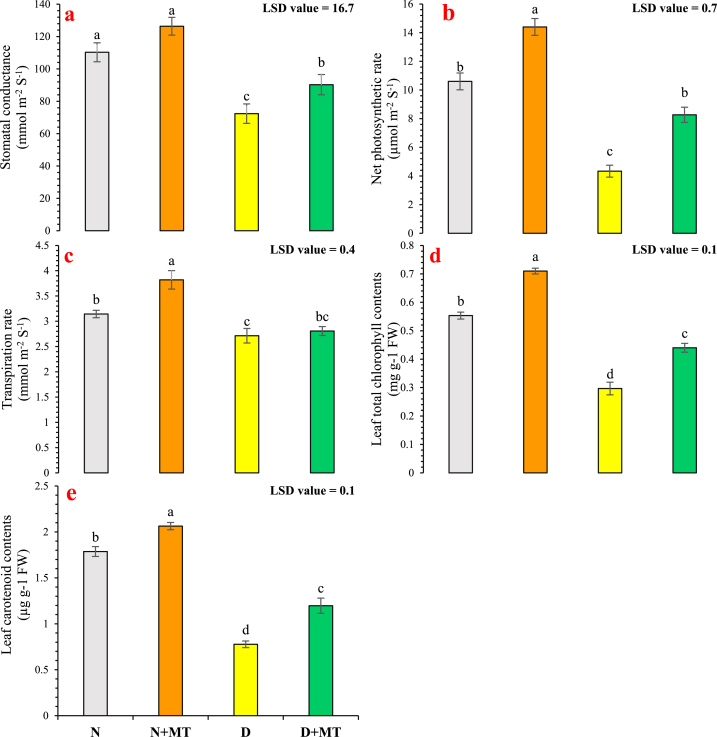
Impact of melatonin on stomatal conductance (**a**), net photosynthetic rate (**b**), transpiration rate (**c**) chlorophyll contents (**d**), and carotenoid contents (**e**) of *Rosa centifolia* under drought-stressed conditions. A mean of six replicates and standard error are displayed in each bar. After using the least significant difference (LSD) test, alphabetical lettering that is different from one another shows statistically significant variations at *p* < 0.05. N = normal water; MT = melatonin; D = drought stress.

Statistically, the impact of water deficiency and melatonin were significant (*p* < 0.05) on all photosynthetic attributes in this study. For *R. centifolia* plants under drought-stress conditions, there were 34.4 %, 59.1 %, and 13.7 % reductions in stomatal conductance, photosynthetic rate, and transpiration rate*,* respectively, compared with well-watered plants. The exogenous foliar melatonin spray increased stomatal conductance by 14.6 % and 24.7 %, photosynthetic rate by 35.8 % and 90.8 %, and transpiration rate by 21.2 % and 3.4 % in normal-watered and drought-stressed conditions, respectively (Fig. 2a–c). The plant pigments showed a highly significant (*p* < 0.01) reduction with water-stressed conditions than normal-water conditions. There were 46.4 % and 56.5 % declines in Chl_t_ and CAR contents, respectively, in leaves of *R. centifolia* plants cultivated under a drought-stressed regime. However, foliar application of melatonin, increases Chl_t_ and CAR concentrations by 28.3 % and 15.5 % with normal water, and by 48.3 % and 54.1 % with drought-stressed regime, respectively (Fig. 2d and e).

### 3.3 Antioxidant enzyme activities, free proline, total soluble protein, and glycinebetaine

**Fig. 3:**
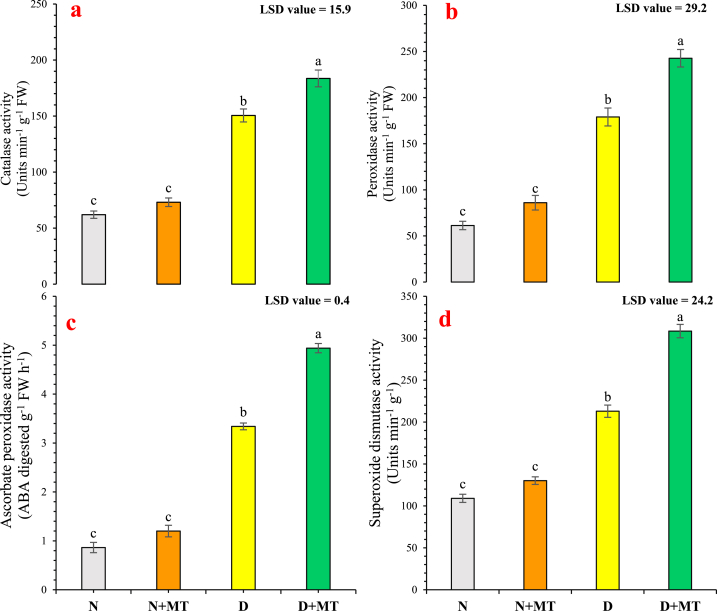
Influence of melatonin on activities of antioxidant enzymes, catalase (**a**), peroxidase (**b**), ascorbate peroxidase (**c**), and superoxide dismutase (**d**) of *Rosa centifolia* leaves under drought-stressed conditions. A mean of six replicates and standard error are displayed in each bar. After using the least significant difference (LSD) test, alphabetical lettering that is different from one another shows statistically significant variations at *p* < 0.05. N = normal water; MT = melatonin; D = drought stress.

**Fig. 4:**
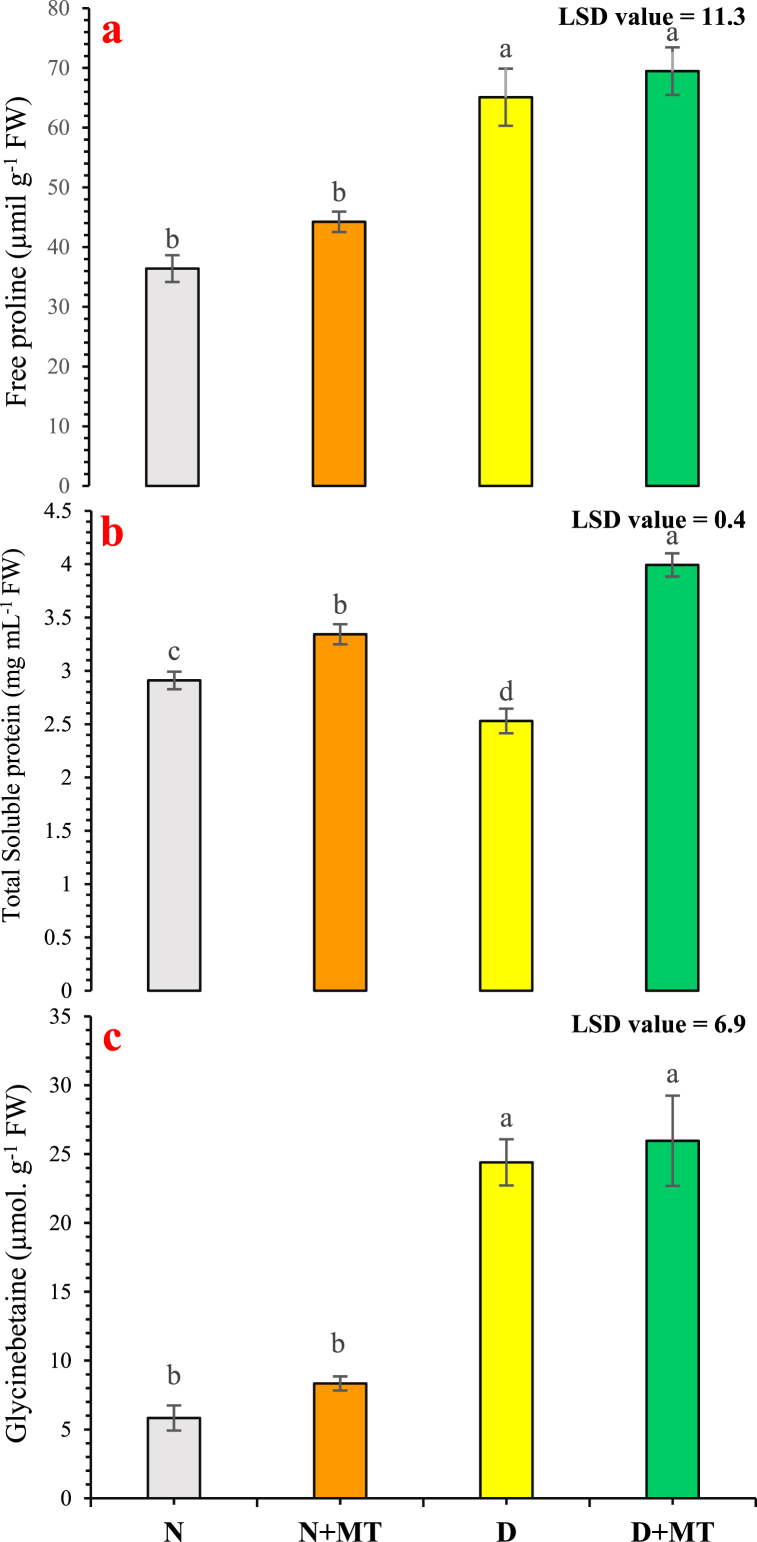
Influence of melatonin application on proline (**a**), total soluble protein (**b**), and glycen betaine (**c**) of *Rosa centifolia* under drought-stressed conditions. A mean of six replicates and standard error are displayed in each bar. After using the least significant difference (LSD) test, alphabetical lettering that is different from one another shows statistically significant variations at *p* < 0.05. N = normal water; MT = melatonin; D = drought stress.

The antioxidant enzymatic activities were highly significantly (*p* < 0.01) increased under water-deficit conditions. At 40 % field capacity, the plants supplemented with melatonin enhance catalase, peroxidase, ascorbate peroxidase, and superoxide dismutase by 21.9 % (Fig. 3a), 35.6 % (Figs. 3b), 47.9 % (Fig. 3c), and 44.8 % (Fig. 3d), respectively, compared with drought-subjected non-treated plants. Free proline contents were highly significantly (*p* < 0.01) increased by 78.8 % under water-deficit (without melatonin spray) *R. centifolia* plants than well-watered plants. The concentrations of proline improved by 21.5 % and 6.7 % due to melatonin foliar application in both normal-watered and water-deficit rose plants, respectively (Fig. 4a). The application of melatonin highly significantly (*p* < 0.01) enhanced total soluble protein and glycinebetaine contents by 14.9 % and 43 %, respectively, under normal-water conditions. However, total soluble protein contents were reduced by 13 %, whereas, glycinebetaine concentrations were enhanced by 318 % in drought-stressed *R. centifolia* plants. Foliar melatonin spray increased protein level by 57.8 % and glycinebetaine level by 6.4 % under drought-stress conditions (Fig. 4b and c).

### 3.4 Malondialdehyde and hydrogen peroxide

**Fig. 5:**
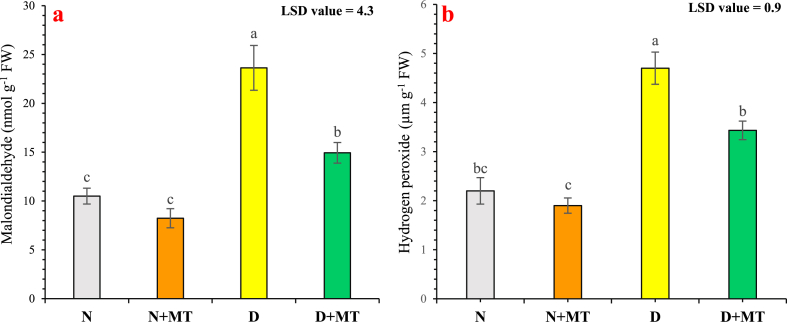
Influence of melatonin application on malondialdehyde (**a**), and hydrogen peroxide (**b**) of *Rosa centifolia* under drought-stressed conditions. A mean of six replicates and standard error are displayed in each bar. After using the least significant difference (LSD) test, alphabetical lettering that is different from one another shows statistically significant variations at *p* < 0.05. N = normal water; MT = melatonin; D = drought stress.

Malondialdehyde contents were highly significantly (*p* < 0.01) decreased after melatonin foliar spray in both drought-stress and normal-water regimes. There was a 36.8 % and 21.6 % reduction in malondialdehyde contents with 40 % field capacity and 80 % field capacity, respectively, due to melatonin foliar supplementation. Meanwhile, drought stress enhanced malondialdehyde level in *R. centifolia* plants by 125 % compared with well-watered conditions (Fig. 5a). Drought stress highly significantly (*p* < 0.01) increased hydrogen peroxide level of rose plants by 113.6 %, compared with normal water conditions. The melatonin supplementation reduced the hydrogen peroxide level by 27 % under a drought-stress regime, and by 13.6 % under normal-water conditions (Fig. 5b).

### 3.5 Anatomical attributes

**Fig. 6:**
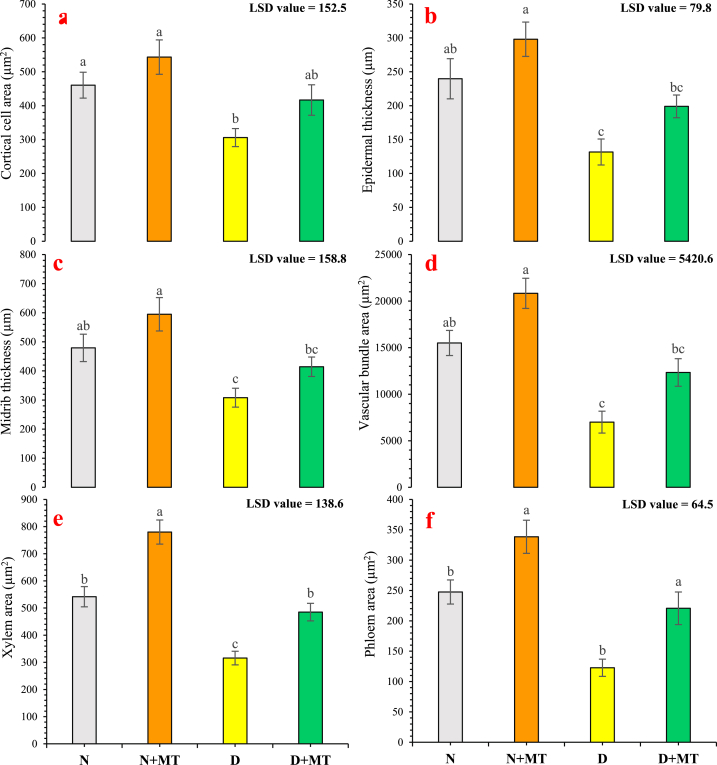
Impact of melatonin on anatomical attributes i.e. cortical cell area (**a**), epidermal thickness (**b**), midrib thickness (**c**), vascular bundle area (**d**), xylem area (**e**), and phloem area (**f**) of *Rosa centifolia* leaves under drought-stressed conditions. A mean of six replicates and standard error are displayed in each bar. After using the least significant difference (LSD) test, alphabetical lettering that is different from one another shows statistically significant variations at *p* < 0.05. N = normal water; MT = melatonin; D = drought stress.

Statistically significant (*p* < 0.05) impact was found on cortical cell area of rose leaves due to drought stress and its mitigation by melatonin. Water-stressed conditions reduced cortical cell area by 33.6 %, compared with the normal-water regime. Whereas, melatonin supplementation enhanced cortical cell area by 18 % (with 80 % field capacity) and by 36.2 % (with 40 % field capacity) in leaves of *R. centifolia* (Fig. 6a). Epidermal thickness of rose leaves was significantly (*p* < 0.05) increased in both normal-water and water-deficit regimes after melatonin spray. There were 24.3 % and 51.1 % increments in epidermal thickness due to melatonin spray in plants cultivated under normal water and drought-stress conditions, respectively. Drought stress (without melatonin treatment) reduced epidermal thickness by 45.1 % compared with the normal-water regime (Fig. 6b). Midrib thickness was significantly (*p* < 0.05) increased with supplementation of melatonin in water-stress and well-water regimes, whereas drought-stress (without melatonin application) reduced midrib thickness by 35.7 %, compared with 80 % field capacity (control). There were 24.1 % and 34.5 % increments in the thickness of midrib with normal-water and water-stress conditions, respectively (Fig. 6c). The foliar supplementation of melatonin significantly increased vascular bundles (by 34.4 % and 76.2 %), xylem (by 44 % and 53.6 %), and phloem area (by 36.7 % and 79.9 %) with 80 % and 40 % field capacity regimes, respectively. Meanwhile, drought-stress conditions remarkably shorten the vascular tissues i.e., vascular bundles, xylem, and phloem area by 54.8 %, 41.7 %, and 50.4 %, respectively, compared with normal water conditions (Fig. 6d–f).

**Fig. 7:**
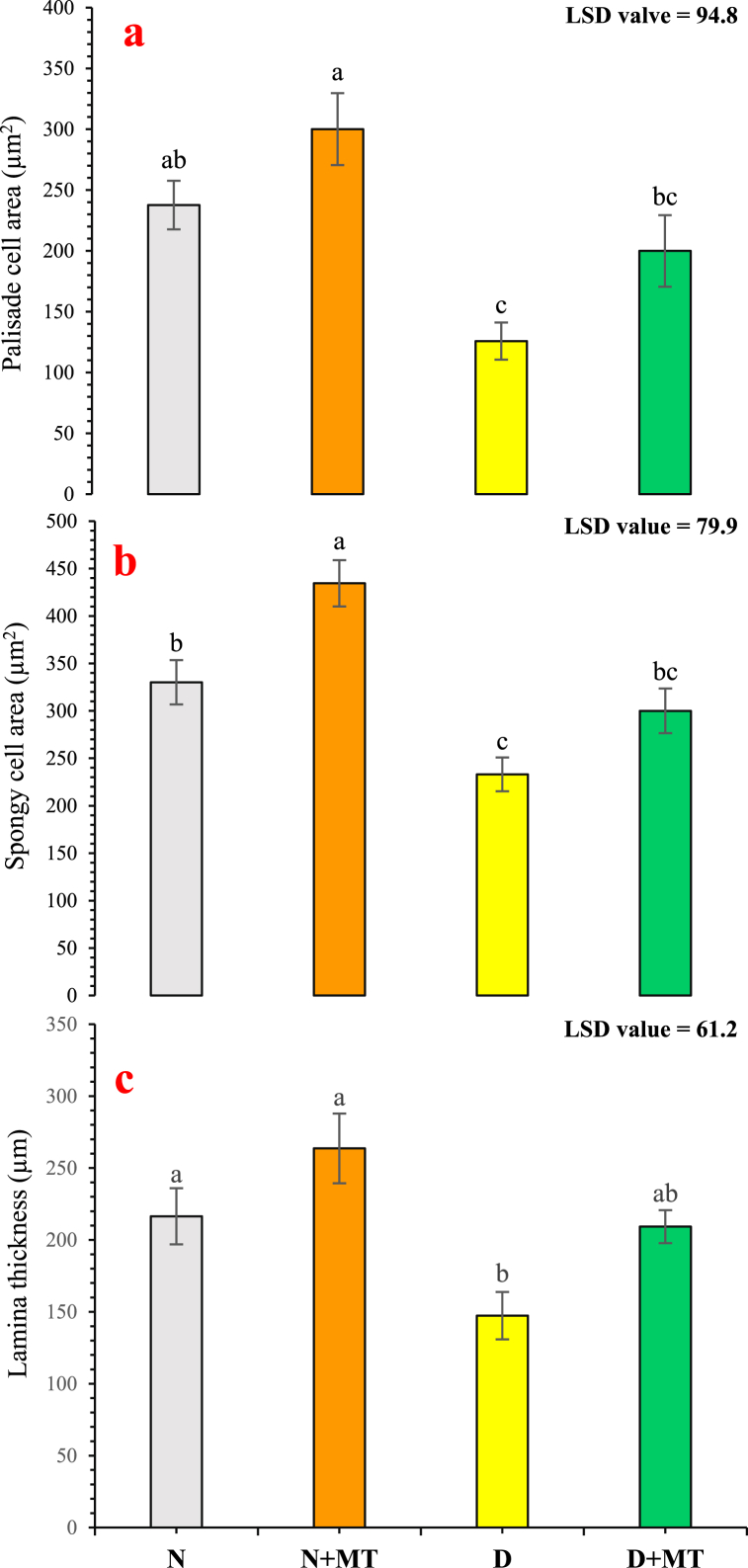
Impact of foliar melatonin supplementation on anatomical attributes like palisade cell area (**a**), spongy cell area (**b**), and lamina thickness (**c**) of *Rosa centifolia* leaves under drought-stressed conditions. A mean of six replicates and standard error are displayed in each bar. After using the least significant difference (LSD) test, alphabetical lettering that is different from one another shows statistically significant variations at *p* < 0.05. N = normal water; MT = melatonin; D = drought stress.

The palisade cells area, spongy cells area, and lamina thickness were significantly (*p* < 0.05) increased, when melatonin was sprayed on *R. centifolia* plants, under drought stress and normal-water conditions. There were increments by 26.3 %, 31.6 %, and 21.8 % under normal-water conditions, and by 58.9 %, 28.7 %, and 42 % under drought-stress conditions, in palisade cells, spongy cells area, and lamina thickness, respectively, due to melatonin foliar sprays. However, these attributes were reduced by 47.1 % (in palisade cell area), by 29.4 % (in spongy cell area), and by 31.9 % (in lamina thickness) under drought stress compared with normal-watered regimes (Fig. 7a–c). Transverse sections of leaf anatomical modifications of *R. centifolia* due to exogenous melatonin spray are also shown in Fig. 1S.

### 3.6 Principal component analysis and pearson correlation heat map

**Fig. 8:**
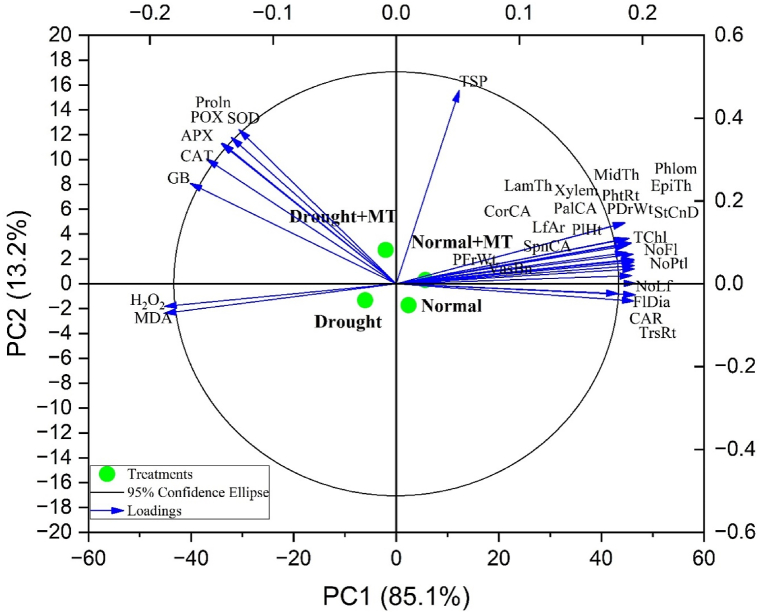
Principal component analysis biplots show correlation amongst different morpho-physiological, biochemical, and anatomical attributes of *R. centifolia* under normal and drought-stress conditions. PlHt = plant height; NoLf = number of leaf; LfAr = leaf area; NoFl = number of flowers; FlDia = flower diameter; NoPtl = number of petals flower^−1^; PFrWt = plant fresh weight; PDrWt = plant dry weight; StCnD = stomatal conductance; PhtRt = photosynthetic rate; TrsRt = transpiration rate; TChl = total chlorophyll; CAR = carotenoid; CAT = catalase; POX = peroxidase; APX = ascorbate peroxidase; SOD = superoxide dismutase; Proln = proline; TSP = total soluble protein; GB = glycinebetaine; MDA = malondialdehyde; H_2_O_2_ = hydrogen peroxide; CorCA = cortical cell area; EpiTh = epidermal thickness; MidTh = midrib thickness; VasBn = vascular bundle area; Xylem = xylem area; Phlom = phloem area; PalCA = palisade cell area; SpnCA = Spongy cell area; LamTh = lamina thickness.

The principal component analysis (PCA) of the studied morpho-physiological, biochemical, and anatomical attributes is presented in Fig. 8. The contributions of the different components are displayed on the x-axis (PC1) and y-axis (PC2). PC1 explained 85.1 % of the total variance, while PC2 accounted for 13.2 %, together representing 98.3 % of the total variance in the dataset. Most of the studied traits exhibited a positive correlation with each other (Fig. 8).

The relationship between drought stress, melatonin application, and specific plant responses was evident. For instance, traits related to oxidative stress, such as malondialdehyde and hydrogen peroxide, were strongly associated with the drought treatment, while antioxidant enzymes (CAT, POX, APX, SOD) and osmoprotectants (Proln, GB) were linked with the drought + melatonin treatment. In contrast, growth-related parameters, including plant height (PlHt), leaf area (LfAr), plant fresh weight (PFrWt), and total chlorophyll content (TChl), showed a positive association with the normal and normal + melatonin treatments, indicating the beneficial role of melatonin in enhancing growth and physiological performance under both normal and drought conditions.

**Fig. 9:**
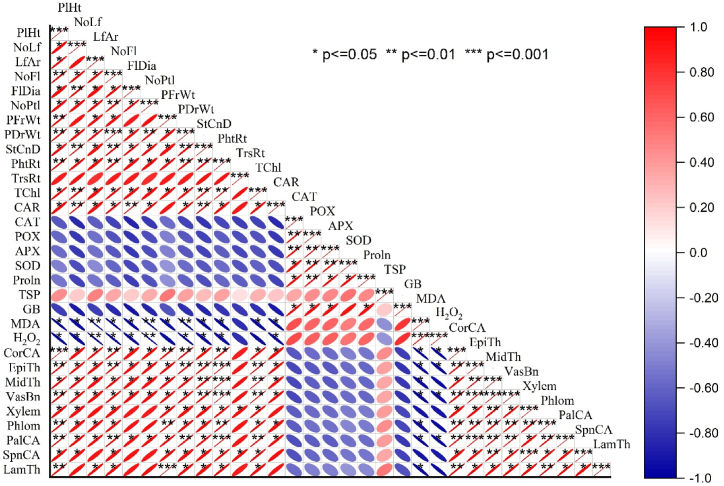
Correlation matrix of different morpho-physiological, biochemical, and anatomical attributes of *R. centifolia* under normal and drought-stress conditions. PlHt = plant height; NoLf = number of leaf; LfAr = leaf area; NoFl = number of flowers; FlDia = flower diameter; NoPtl = number of petals flower^−1^; PFrWt = plant fresh weight; PDrWt = plant dry weight; StCnD = stomatal conductance; PhtRt = photosynthetic rate; TrsRt = transpiration rate; TChl = total chlorophyll; CAR = carotenoid; CAT = catalase; POX = peroxidase; APX = ascorbate peroxidase; SOD = superoxide dismutase; Proln = proline; TSP = total soluble protein; GB = glycinebetaine; MDA = malondialdehyde; H_2_O_2_ = hydrogen peroxide; CorCA = cortical cell area; EpiTh = epidermal thickness; MidTh = midrib thickness; VasBn = vascular bundle area; Xylem = xylem area; Phlom = phloem area; PalCA = palisade cell area; SpnCA = Spongy cell area; LamTh = lamina thickness. The color gradient showed the direction and strength of the correlations, with red color showing positive correlations and the blue color showing negative correlations among different characteristics of *R. centifolia*. ∗p ≤ 0.05, ∗∗p ≤ 0.01, ∗∗∗p ≤ 0.001.

The correlation matrix (Fig. 9) highlights significant relationships between various morpho-physiological, biochemical, and anatomical traits of *R. centifolia* under normal and drought-stress conditions. Among the morpho-physiological traits, plant height (PlHt) shows strong positive correlations with number of leaves (NoLf) (r = 0.89, p < 0.001) and leaf area (LfAr) (r = 0.91, p < 0.001). Similarly, number of flowers (NoFl) is highly correlated with flower diameter (FlDia) (r = 0.85, p < 0.001) and number of petals (NoPtl) (r = 0.83, p < 0.001), indicating that taller plants with more flowers tend to have larger flowers with more petals. In terms of physiological parameters, photosynthetic rate (PhtRt) is positively correlated with transpiration rate (TrsRt) (r = 0.75, p < 0.001) and stomatal conductance (StCnD) (r = 0.78, p < 0.001), highlighting a coordinated gas exchange regulation. Total chlorophyll (TChl) correlates with carotenoid (CAR) (r = 0.72, p < 0.001) and catalase (CAT) (r = 0.65, p < 0.01), linking higher chlorophyll content to oxidative stress protection. Biochemically, proline (Proln) and glycinebetaine (GB) show a strong positive correlation (r = 0.81, p < 0.001), both acting as osmoprotectants. Total soluble proteins (TSP) also positively correlate with proline (r = 0.78, p < 0.001). Moreover, malondialdehyde (MDA) is positively correlated with hydrogen peroxide (H2O2) (r = 0.68, p < 0.01), indicating increased oxidative stress. At the anatomical level, vascular bundle area (VasBn) correlates with xylem area (r = 0.83, p < 0.001) and midrib thickness (MidTh) (r = 0.80, p < 0.001), suggesting well-developed vascular tissues enhance water transport under stress (Fig. 9).

## 4 Discussion

Water availability is the most vital factor in the successful cultivation of plants, especially those with shallow root systems, including rose [4]. The plants developed different morphological, physiological, and biochemical responses to combat drought tolerance [38]. Melatonin is a pleiotropic bio-stimulant molecule that can alleviate drought stress and enhance crop quality [39]. In this study, drought stress noticeably reduced morphological attributes such as plant height, number of leaves and flowers, and petal biomass of *R. centifolia* (Fig. 1). This growth reduction under drought-stressed conditions may be the morphological response of plants to prevent loss of water by dropping the leaves for lower transpiration [10]. Additionally, this reduction may be due to the lethal impacts of drought on the uptake of water as well as nutrients and might decrease the cell division and expansion, resulting in a decline in the plant height, leaf number, and area [40,41]. Under drought conditions, osmotic stress activates, which hampers cell elongation and ultimately decreases plant morphological traits [42]. Langaroudi et al. [43] stated that a reduction in plant morphological attributes, lessens carbon assimilation, and degrades chlorophyll pigments and photosynthesis, affecting nucleic acid metabolism and protein synthesis, resulting in reduced vegetative and reproductive plant growth attributes. The previous experiments reported that melatonin can enhance the morphological traits of various horticultural crops [[44], [45], [46]]. Melatonin is a hormone that has been proven to have dual roles in the plants such as growth elevation and protection against abiotic stresses [10,42]. Likewise, melatonin supplementation improves cell elongation, and shoot and leaf water contents, decreases osmotic stress, and enhances the activities of antioxidant enzymes. The present study also showed increased flower yield and quality with melatonin supplementation under both drought and normal water conditions. Similar results were obtained by Arnao and Hernandez-Ruiz [47] who stated that melatonin in trace amounts enhances the flowering of *Arabidopsis thaliana*. Melatonin directly interacts with several reactive oxygen species, neutralizing them before they can cause cellular injury. It can increase the antioxidant enzyme activities endogenously, thereby improving the intrinsic defense systems of the cells. Plants cultivated under drought stress followed by melatonin spray had less impairment of the morphological growth attributes and remarkably lower inhibition of photosynthetic processes, activities of antioxidant enzymes, and reduced levels of oxidative stress markers compared to the plants cultivated under drought stress conditions (Fig. 1, Fig. 2, Fig. 3, Fig. 5). Melatonin-induced elevation in drought tolerance could be because melatonin normalizes stomatal behavior under stress conditions, increasing the availability of internal CO_2_ concentrations while enhancing the corboxylation process, carbonic anhydrase activity, and photosynthetic efficiency [48]. Lezoul et al. [17] reported that melatonin supplementation increased water relations and higher petal biomass incarnation. Furthermore, melatonin-induced stomatal closure in roses during photosynthesis may contribute to a decrease in moisture loss and maintaining the flower biomass [49]. These results explain the better growth and higher biomass accumulation in *R. centifolia* plants treated with melatonin.

Photosynthesis is the most vital physiological mechanism directly associated with plant growth and dry matter production. Photosynthetic activities mainly depend on the stomatal status and movement of water by the conductive tissues [50]. Melatonin averts the degradation of chlorophyll molecules under limited water conditions and increases stomatal conductance, transpiration, and photosynthesis [51]. In our experiment, increased net photosynthetic rate, stomatal conductance, and transpiration rate in drought-stressed rose plants were defined as a metabolic stimulus in plants by melatonin (Fig. 2). These findings could be clarified by the retaining the chlorophyll contents, which allowed the conservation of light-use efficiency. This may be mediated by the prevention of chlorophyll degradation and the subsequent synthesis of porphyrins by the proper D-aminolevulinate synthase [52]. Chlorophyll degradation is catalyzed by enzymes i.e. pheophytinase, chlorophyll degrading peroxidase, and chlorophyllase [53]. The decline in chlorophyll degradation after melatonin supplementation is due to the down-regulation of pheophytinase, chlorophyll degrading peroxidase, and chlorophyllase genes [54]. Similar findings were reported in melatonin-treated young *Carya cathayensis* plants exposed to various drought stress levels. The protection of photosynthetic mechanisms can justify the enhanced biomass accrual as flower diameter and petal biomass of roses. These melatonin-mediated impacts enable plants to continue water absorption and distribution and eventually result in drought stress tolerance in plants. The temperature and water status of the leaf is positively regulated by the transpiration rate. The enhanced rate of transpiration by melatonin empowers the plants to uphold a lesser leaf temperature, thus enhancing photosynthetic efficiency [55]. The positive regulatory role of melatonin on stomatal conductance and transpiration rate via the regulation of abscisic acid concentration was also reported in pepper [56], and tomato [57]. Chang et al. [58] found that melatonin helps to uphold the integrity of D1 protein (a key component of PSII protein), thereby improving the efficiency and the rate of photosynthesis. Furthermore, melatonin also recovers the concentrations of photosynthetic accessory pigments, like chlorophyll *b*, carotenoids, and anthocyanin under water-shortage conditions [59,60].

Chlorophylls and carotenoids are two kinds of photosynthetic pigments in plants. Chlorophyll contents are vital for absorbing and transmitting light energy into useful chemical energy at the photoreaction center, while carotenoids act as photo-protectants, dissipating excess energy before they may damage the plant cells [61,62]. Melatonin can alleviate the reduction of chlorophyll and carotenoid contents and improve plant growth under drought stress [63]. Analogous results were found from this study (Fig. 2). Jahan et al. [55] reported that melatonin remarkably up-regulated the expression of chlorophyll oxygenase, and protochlorophyllide oxidoreductase genes, and enhanced chlorophyll contents under oxidative stress, in agreement with our results. The reduction in photosynthetic pigments might be associated with the decrease in cysteine and methionine contents (key constituents of chloroplast target protein) under drought stress [6]. These findings indicated that exogenous melatonin enhances the photoprotective ability and light-harvesting capacity by elevating the total chlorophyll level and carotenoid synthesis and metabolism, thus improving the drought tolerance of *R. centifolia* plants.

Antioxidative enzymes play a vital role in stress tolerance. Increased activities of antioxidant enzymes lead to potential and specific reactive oxygen species scavenging under different stressful conditions [10]. The catalase, superoxide dismutase, peroxidase, and ascorbate peroxidase as major antioxidant enzymes were remarkably improved in drought-stressed rose plants compared with normal water conditions (Fig. 3). These findings might be due to the over-accumulation of antioxidants to alleviate the oxidative injury and scavenge reactive oxygen species in *R. centifolia* plants under water deficit conditions [64]. Similar findings were reported in sugar beet [65], sword lily [66], and potato [67]. Melatonin is a multi-functional regulatory molecule and is regarded as a universal antioxidant [67], due to its strengthening ability to antioxidant defense system and increases stress tolerance in plants, mostly by detoxifying excess reactive oxygen species, induced by abiotic stresses [69]. Altaf et al. [10] showed that stressed plants treated with 100 μM melatonin significantly increased antioxidant enzyme activity of ascorbate peroxidase, catalase, peroxidase, and superoxide dismutase and their relative gene expression. Arnao and Hernandez-Ruiz [69] stated that the key function of melatonin is to raise the efficiency of antioxidant enzymes in plants. In this regard, it has been confirmed that melatonin foliar spray under drought-stressed conditions enhanced antioxidant enzyme activity in a variety of plant species, including tomato [70], Sweet cherry [71], and rose [49]. Furthermore, SOD plays a major role in reactive oxygen species scavenging and converting the $O_{2}^{-}$ to O_2_ and H_2_O_2_, after that POD and CAT break down the H_2_O_2_ to H_2_O [42]. The supplementation of melatonin during the drought period, decreased oxidative damage and reestablished damaged cellular membranes, reduced reactive oxygen species buildup by scavenging reactive oxygen species, improved antioxidant enzymatic activities, and ultimately promoted plant growth [72]. The exact mechanism by which melatonin increases the production of enzymes needs further experiments; as it may be due to direct interactions with already-existing enzymes or to signal transduction pathways that control gene expression.

Drought-stressed plants store appropriate solutes, such as proline, to aid the absorption of water, raise the osmotic potential of the plants, and lessen cellular damage [73]. Zulfiqar et al. [66] found elevated proline levels in salt-stressed *Gladiolus grandifloras* plants, which may help to withstand salt stress by antioxidative and osmoregulative defenses. Proline buildup is a general marker of drought tolerance and permits osmotic modification which results in evading dehydration of cells [72]. Melatonin increases proline contents in tissues under stress conditions to tackle oxidative stress [40]. Proline contents in drought-stressed rose plants increased to some extent when melatonin was exogenously supplemented (Fig. 4a). Under abiotic stress, proline helps to prevent damage to the cell membrane, DNA, and protein by scavenging OH^•^ radicals and quenching singlet oxygen [68]. Accordingly, an increment in proline levels in drought-stressed plants with the treatment of melatonin was observed, resulting in an elevation in the uptake of water, boosting the photosynthesis systems, and finally improved flowering attributes in drought-stressed plants [74]. In our present study, the total soluble protein contents were noticeably boosted with the supplementation of melatonin in drought-stressed and normally watered *R. centifolia* plants (Fig. 4b). The elevation in soluble protein contents is a supreme mechanism for the alleviation of drought-stressed damage by melatonin [75]. Melatonin augmented protein contents in *chrysanthemum* plants, resulting in raised photosynthetic traits and antioxidant capacity under drought [74]. Jinxiang et al. [76] reported that exogenous melatonin mitigates the root DNA injury in *Ardisia crenata* under lead stress and increases the protein folding capability of cells. Melatonin supplementation showed overexpression of ionic transport proteins *NHX1* (Na^+^/H^+^ exchanger) and *AKT1* kinase during exposure to abiotic stresses [77]. Similar results were obtained by Luo et al. [73], who reported that melatonin increases osmotic regulation by enhancing proline and total soluble protein contents, regulating non-enzymatic (glutathione and ascorbic acid) and enzymatic (catalase, peroxidase, and superoxide dismutase) antioxidants. To combat the lethal impacts of reactive oxygen species, plants initiate a defense response that includes the synthesis of solutes such as glycinebetaine to withstand dehydration and oxidative stress [78]. In our study, supplementation of melatonin enhanced the glycinebetaine levels in drought-stressed rose plants (Fig. 4c). The raised concentrations of glycinebetaine in plants may be due to various mechanisms such as detoxification of reactive oxygen species, osmotic adjustment, and the integrity of membrane in plants under water-deficit conditions [79]. Glycinebetaine protects the photosynthetic machinery by stabilizing the repaired protein activity and reducing lipid peroxidation under severe oxidative stress conditions [80].

Lipid peroxidation (malondialdehyde) and hydrogen peroxide levels as stress indicators have been used as oxidative injury biomarkers. In the present study, oxidative damage levels, as measured by malondialdehyde and hydrogen peroxide were drastically increased in rose plants cultivated under drought stress treatment. These findings might be because drought stress causes lipid peroxidation and oxidative damage for plant organelles, primarily plasma membrane, mitochondria, and chloroplast, resulting in an elevation in lipid peroxidation and hydrogen peroxide [64]. Conversely, melatonin supplementation efficiently protected plant cells from oxidative injury (Fig. 5). In previous studies, Altaf et al. [10], Al-Shammari et al. [64], and Naghizadeh et al. [41] showed a remarkable lowering of malondialdehyde and hydrogen peroxide by melatonin in tomato, soya bean, and basil plants, respectively, under drought stress. Similarly, Sandoval et al. [81] revealed that melatonin application tremendously reduced the levels of malondialdehyde and hydrogen peroxide contents in blueberry plants due to increased metabolite production, reduction in oxidative stress, and repairing of interrupted cellular membrane, improved photosynthesis, therefore decreasing contents of oxidative stress biomarkers.

Anatomical characteristics of different plant parts are key indicators under drought stress. Plants have developed various adaptation strategies to adjust to their immediate environment [82]. In this study, drought stress adversely affected the cortical cell area, epidermal and midrib thickness, vascular bundles, and mesophyll tissues of *R. centifolia* leaves (Fig. 6). However, melatonin supplementation significantly ameliorated the harsh drought impact and improved anatomical features of roses under water-deficit conditions (Fig. 6). A specific anatomical modification such as leaf lignification may contribute to water conservation and drought stress tolerance [83]. Moustafa-Farag et al. [84] elaborated that melatonin application increased leaf lignification through increased epidermal thickness and vascular tissues in roses under drought-stressed conditions. Melatonin significantly improved the thickness of the cortex, midrib, epidermis, and vascular bundles in rose leaves (Fig. 6). A well-organized and developed vascular tissue can be considered as a perilous structural modification under water-limited conditions [85]. Drought stress reduces CO_2_ absorption in plant cells by limiting diffusion through the stomata and decreases the lamina thickness, which decreases the level of CO_2_ in the mesophyll through changes in carbon metabolism and leaf photochemistry [86]. Tan et al. [87] reported that exogenous melatonin supplementation increased the stomatal area and thickness of vascular bundles in young leaves of cabbage. Similarly, Yan et al. [77] reported that melatonin-treated plants possessed thicker leaves with improved vascular bundle areas and osmolyte accumulation in *Carex leucochlora* plants under drought stress. Melatonin-treated plants under drought-stressed conditions increased the spongy cell area, resulting an increment in the size of the palisade and spongy cell areas (Fig. 7). These tiny mesophyll cells deliver a remarkable resistance against cell damage under abiotic stress conditions [11]. These findings suggest that melatonin treatment preserves leaf anatomical features to lessen photosynthetic inhibition and has protective impacts against drought-mediated damage on morpho-physiological, biochemical, and anatomical attributes of *R. centifolia* plants. These findings partially fill the gap by seeking the impact of exogenous melatonin supplementation on the major melatonin-mediated physiological, biochemical and anatomical modifications influencing rose production under drought-stressed conditions.

## 5 Conclusions

The results of this study confirmed that melatonin significantly improved growth by inducing drought tolerance in *R. centifolia* plants. Positive impacts of exogenous melatonin application on morphological characteristics such as the number of leaves and flowers, and the petal biomass were recorded to be associated with melatonin-mediated enhancement in photosynthetic pigments and stomatal conductance levels. Melatonin reduced malondialdehyde and hydrogen peroxide levels through stimulation of antioxidant enzyme activities, total soluble proteins, and free proline contents under drought-stress conditions. Furthermore, leaf anatomical structures including vascular bundles, mesophyll, and epidermal tissues of rose plants were improved with melatonin supplementation. These results highlight melatonin potential as a sustainable, and eco-friendly method for enhancing the quantity and quality of ornamental crops in stress-prone regions. For future research, it is strongly suggested that the role of melatonin against different abiotic stresses under field conditions should be investigated, as melatonin impacts are mostly studied under controlled or limited conditions. Some detailed molecular approaches are necessary, to unveil the potential mechanisms of melatonin-induced abiotic stress tolerance in ornamental plants, commercially used for cosmetics and pharmaceutical purposes. Additionally, the development of crop varieties with altered melatonin signaling will certainly give rise to new standards for melatonin supplementation in various ornamental crops in a changing environment.

## CRediT authorship contribution statement

**Muhammad Ahsan:** Writing – original draft, Project administration, Formal analysis, Data curation, Conceptualization. **Adnan Younis:** Validation, Supervision, Formal analysis. **Aftab Jamal:** Writing – review & editing, Software, Resources, Methodology. **Mohammed O. Alshaharni:** Resources, Project administration, Funding acquisition, Formal analysis. **Uthman Balgith Algopishi:** Writing – review & editing, Validation, Resources, Funding acquisition. **Abeer Al-Andal:** Validation, Resources, Investigation, Formal analysis. **Mateen Sajid:** Resources, Methodology, Formal analysis, Conceptualization. **Muhammad Naeem:** Visualization, Validation, Project administration, Investigation, Formal analysis. **Jawad Ahmad Khan:** Validation, Resources, Formal analysis. **Emanuele Radicetti:** Writing – review & editing, Writing – original draft, Supervision, Software, Resources, Methodology, Investigation, Formal analysis. **Mohammad Valipour:** Writing – review & editing, Writing – original draft, Visualization, Validation. **Gulzar Akhtar:** Validation, Resources, Formal analysis, Data curation.

## Funding

This research work was funded by the Deanship of Scientific Researcher Project (RGP2/56/45) King Khalid University, Saudi Arabia.

## Declaration of competing interest

The authors declare that they have no known competing financial interests or personal relationships that could have appeared to influence the work reported in this paper.
