# Supplementary material for: Melatonin induces drought stress tolerance by regulating the physiological mechanisms, antioxidant enzymes, and leaf structural modifications in Rosa centifolia L
Source: Heliyon. 2024 Dec 17;11(1):e41236. doi: 10.1016/j.heliyon.2024.e41236 (PMC11729657; doi:10.1016/j.heliyon.2024.e41236)
Supplement: Multimedia component 1 [file mmc1.docx]

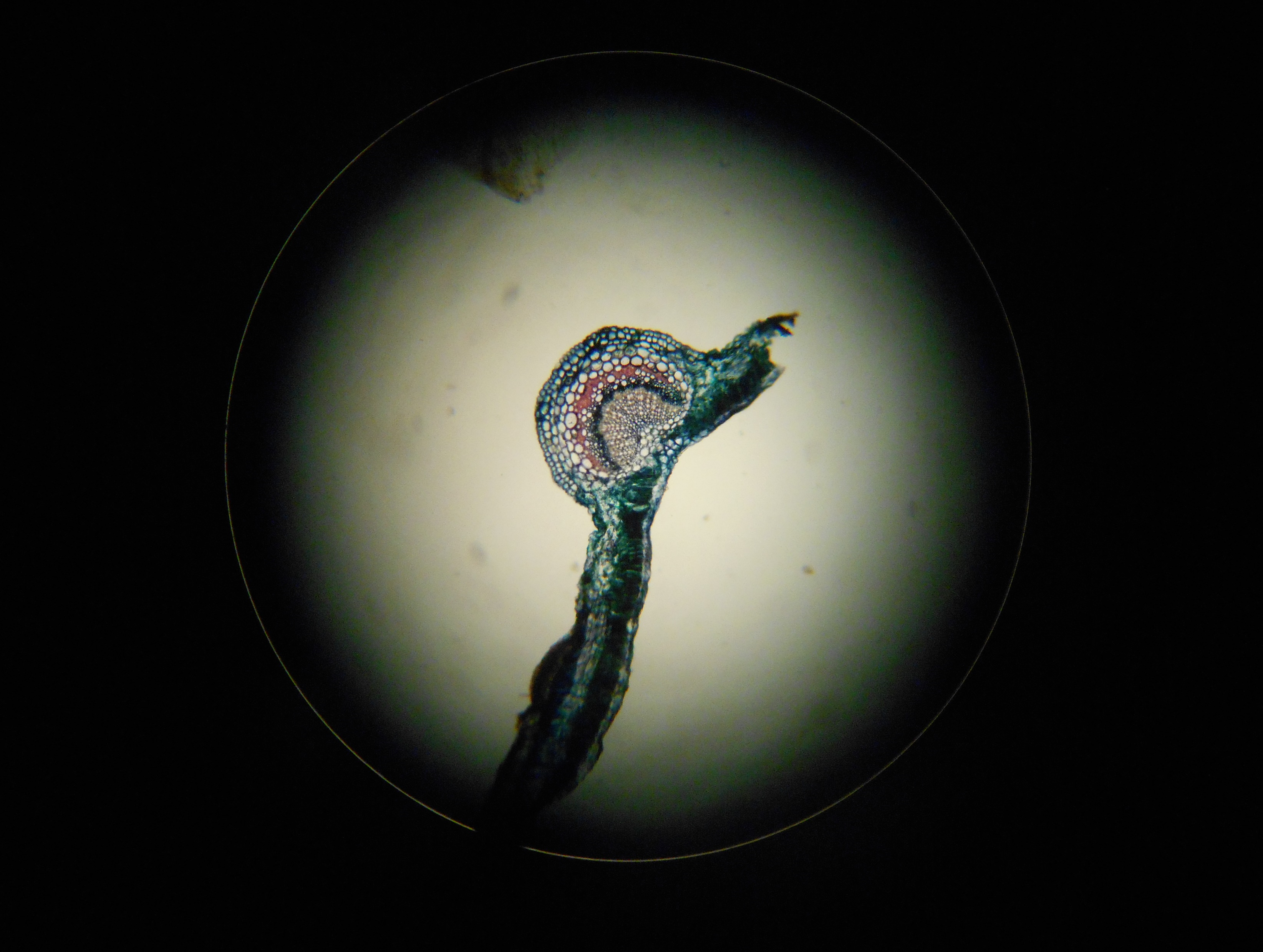

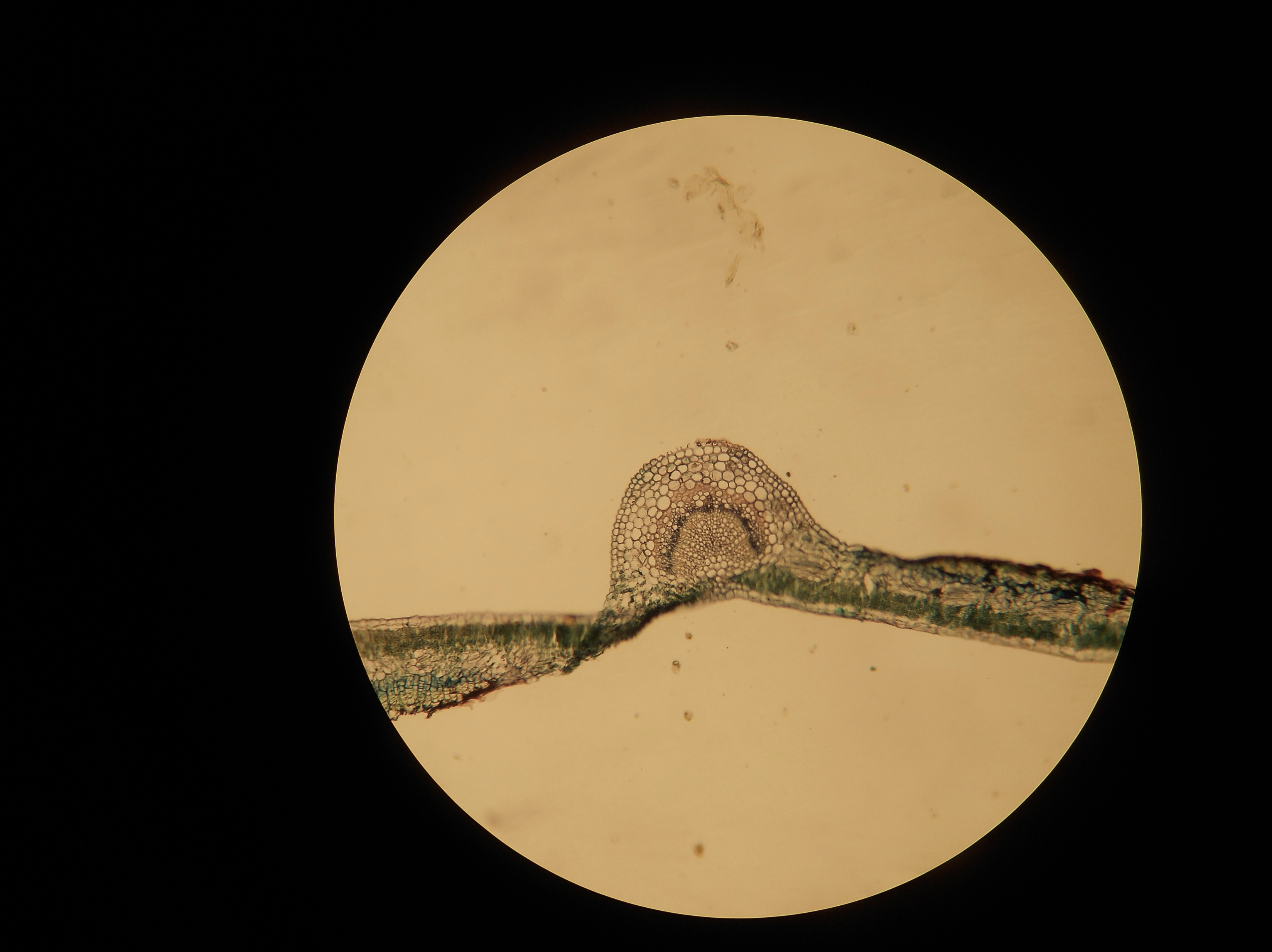


**b**

**a**

**d**

**c**


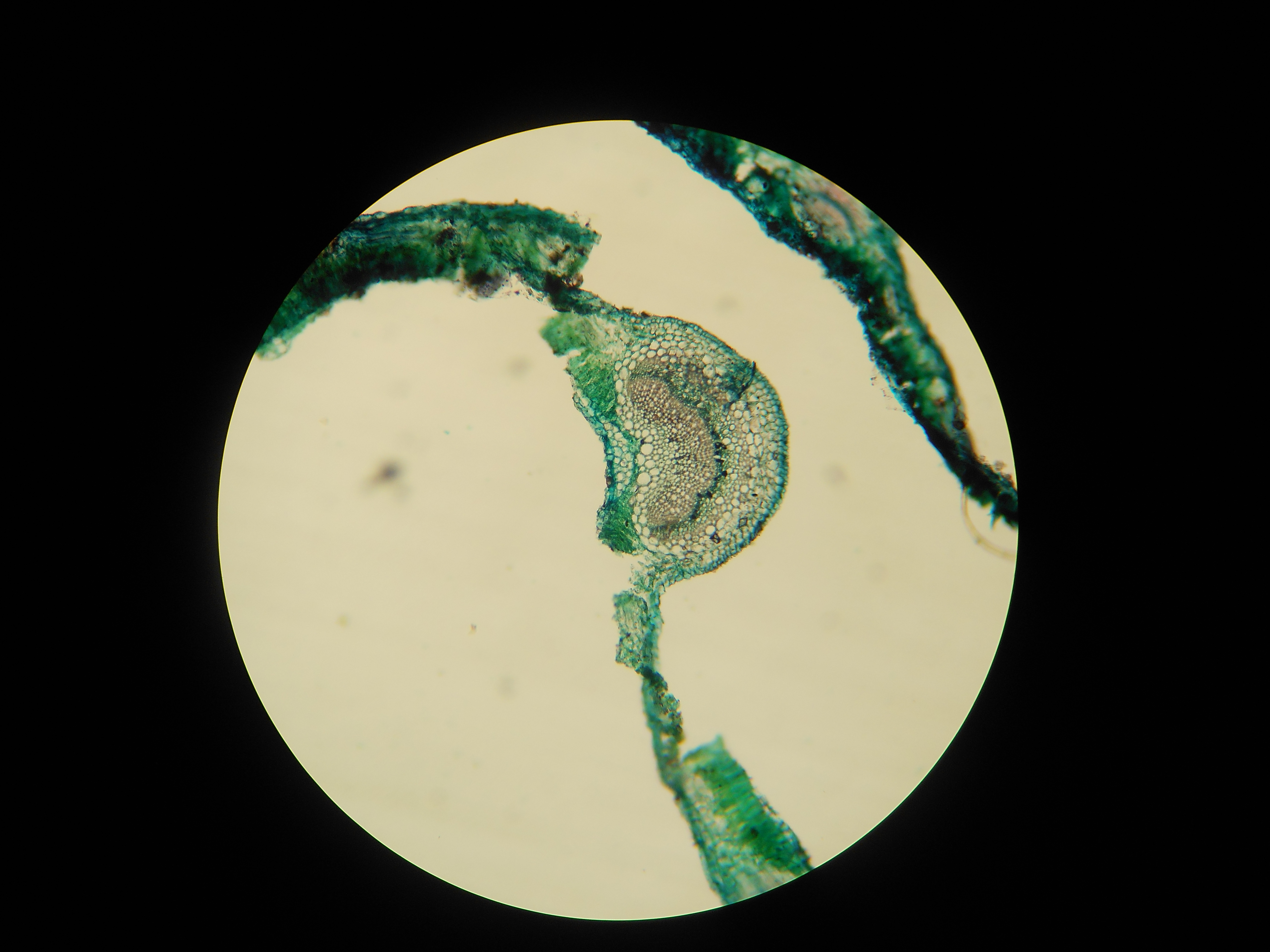

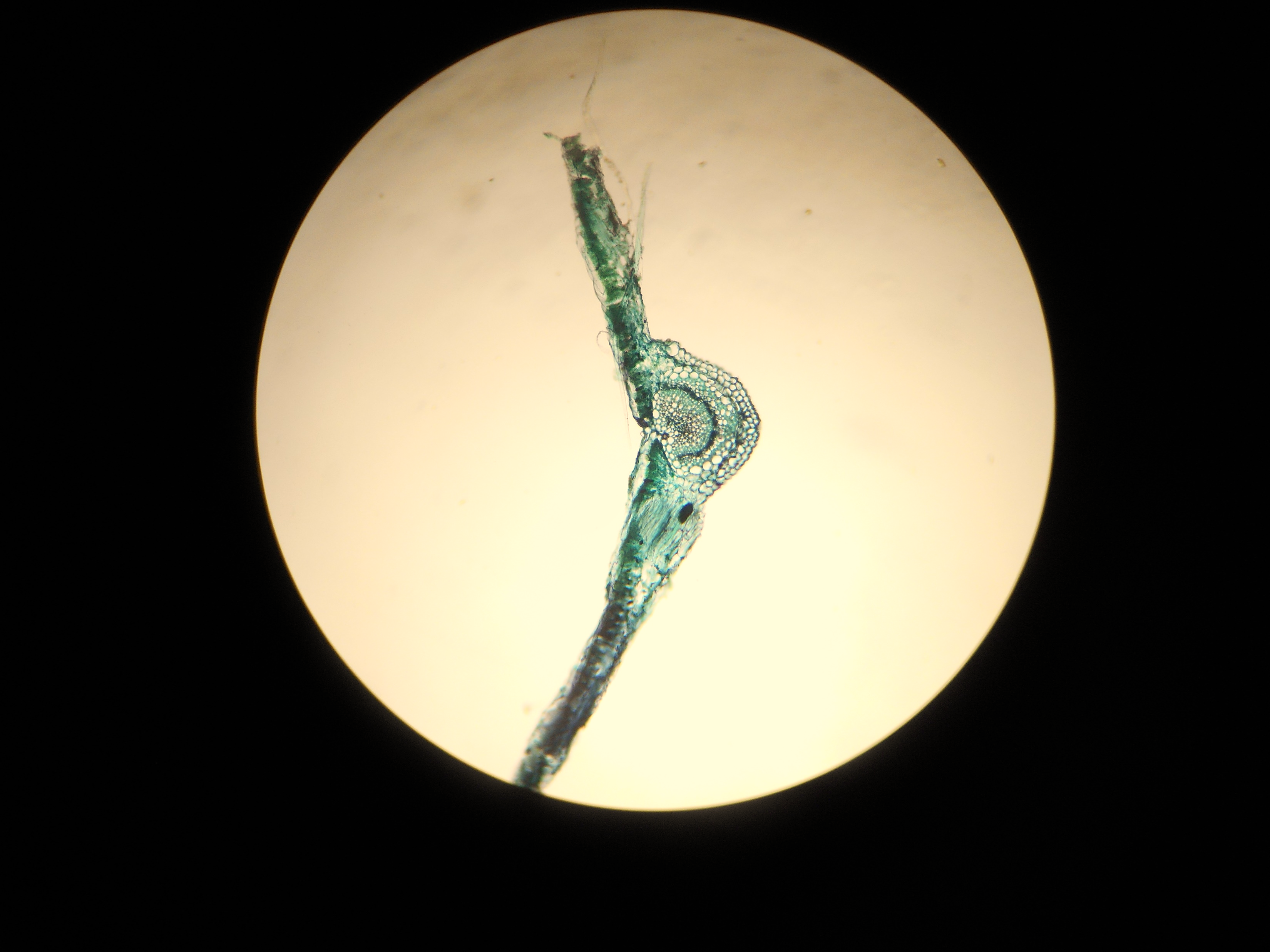


**SC**

**Ph**

**ET**


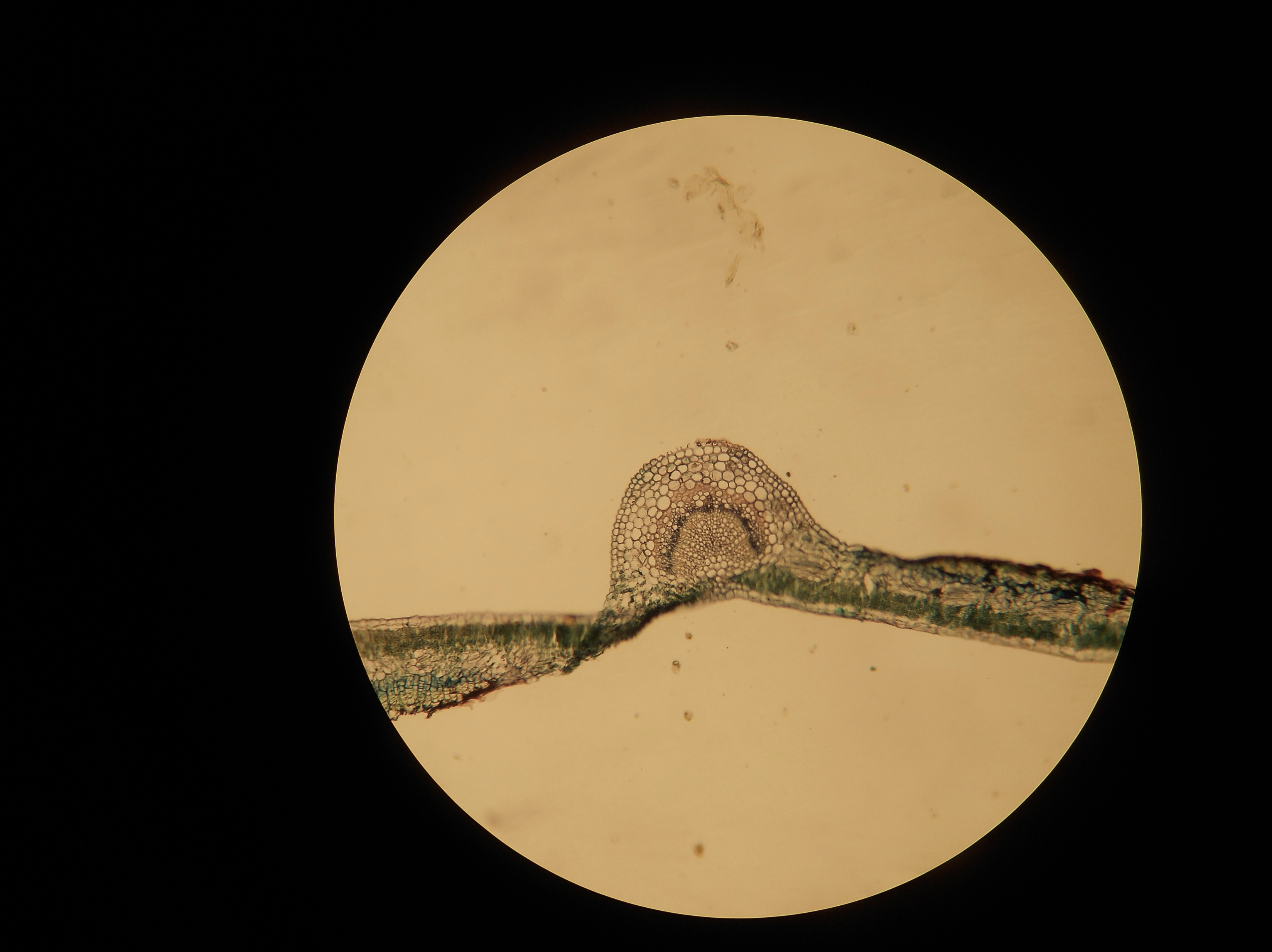

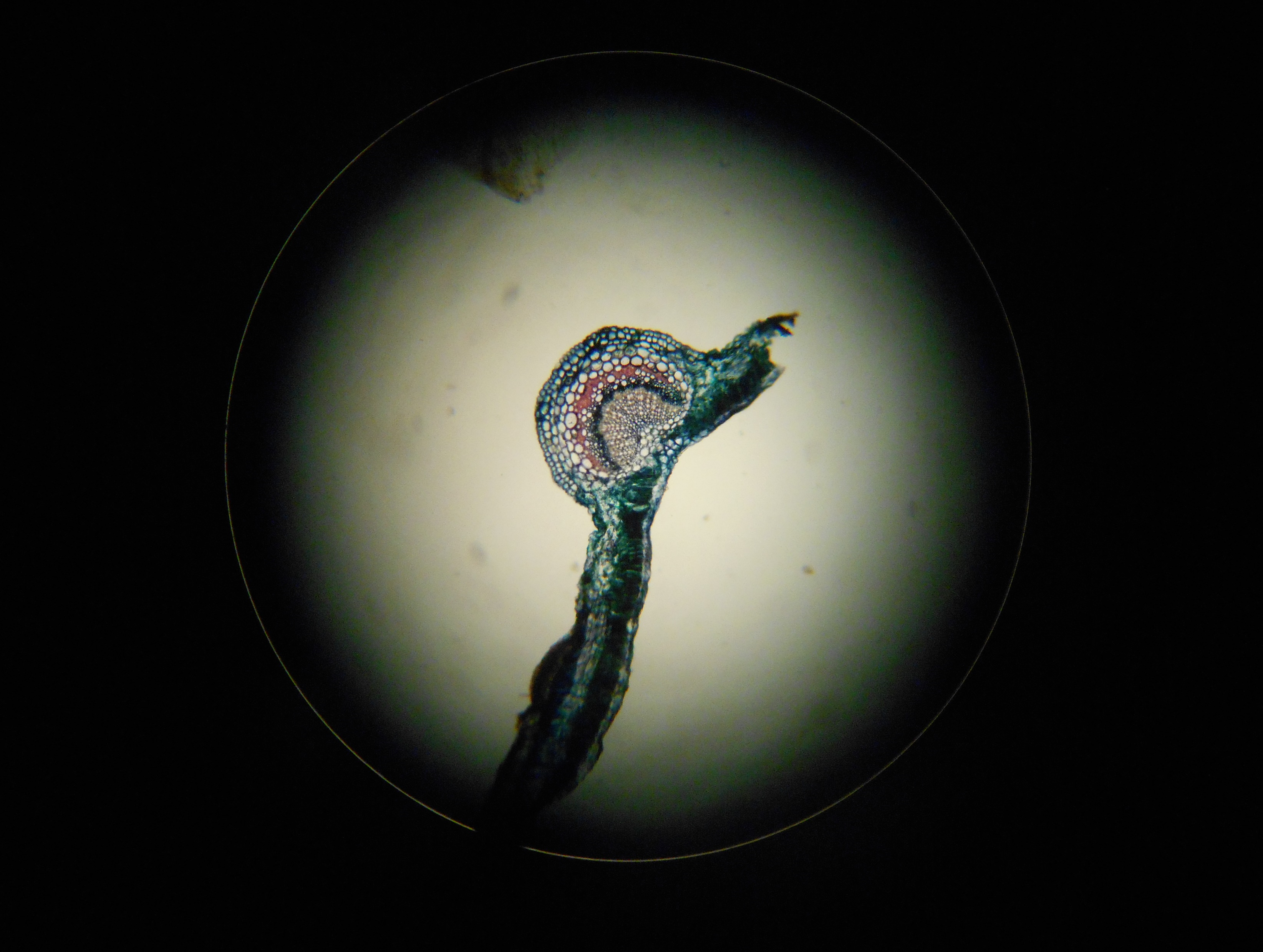

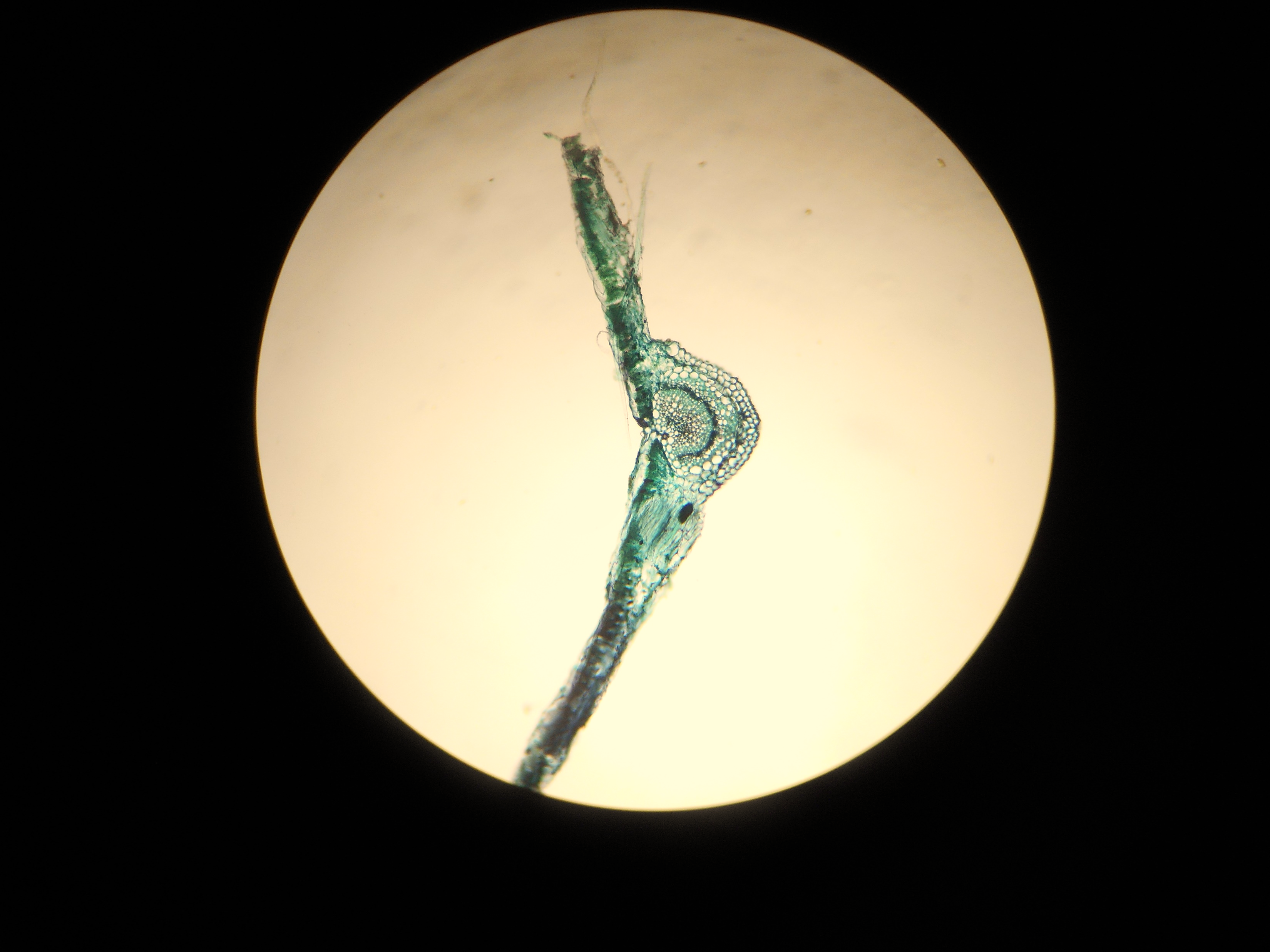

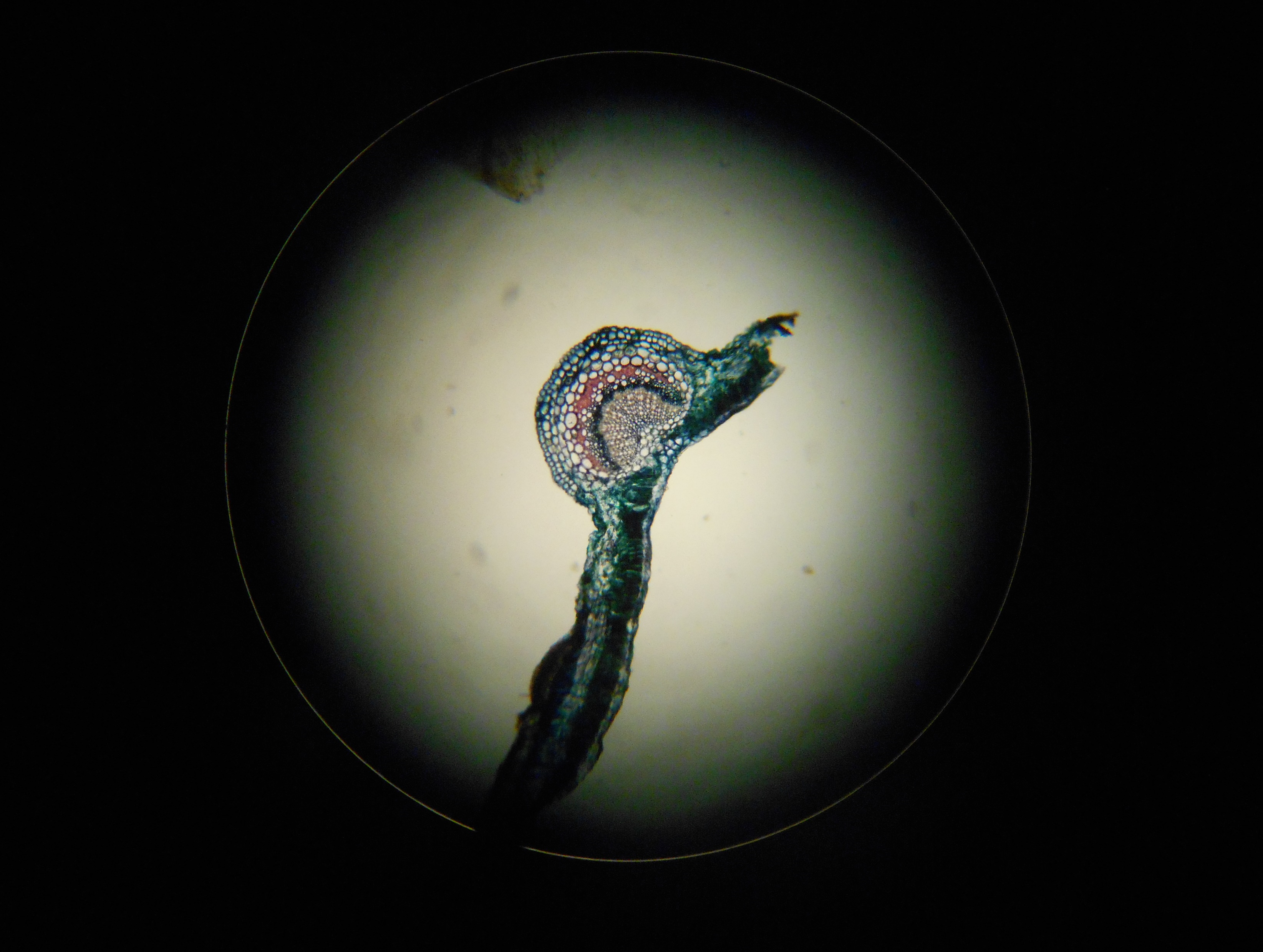


**CC**

**A**

**B**

**D**

**D**

**MT**

**Xy**

**Pa**

**D+MT**

**D**

**N+MT**

**N**

**Fig. S1:** Transverse section of leaf anatomical modifications of *Rosa centifolia* due to melatonin supplementation. N = normal conditions with 80% field capacity (**a**), N+MT = melatonin spray under normal conditions (**b**), D = drought stress conditions with 40% field capacity (**c**), D+MT = melatonin spray under drought stress (**d**). ET = Epidermal thickness; CC = Cortical cells; Xy = Xylem; MT = Midrib thickness; Ph = Phloem; SC = Spongy cells; Pa = Palisade cells.
